# Magnitude and associated factors of aggressive behaviour among patients with bipolar disorder at Amanual Mental Specialized Hospital, outpatient department, Addis Ababa, Ethiopia: cross-sectional study

**DOI:** 10.1186/s12888-016-1151-8

**Published:** 2016-12-12

**Authors:** Habte Belete, Haregwoin Mulat, Tolesa Fanta, Solomon Yimer, Takele Shimelash, Tilahun Ali, Tilahun Tewabe

**Affiliations:** 1 Psychiatry Department, College of Medicine and Health Science, Bahir Dar University, Bahir Dar, PO box 79, Ethiopia; 2 Psychiatry Department, College of Medicine and Health Science, University of Gondar, Gondar, Ethiopia; 3 Amanual Mental Specialized Hospital, Addis Ababa, Ethiopia; 4 Psychiatry Department, College of Medicine and Health Science, Dilla University, Dilla, Ethiopia; 5 Felegehiwot Referral Hospital, Bahir Dar, Ethiopia; 6 Psychiatry Department, College of Medicine and Health Science, Haromaya University, Harrer, Ethiopia; 7 Nursing Department, College of Medicine and Health Science, Bahir Dar University, Bahir Dar, Ethiopia

**Keywords:** Aggressive behaviour, Bipolar disorder, Ethiopia

## Abstract

**Background:**

Aggressive behavior is a challenging behavior among bipolar patients that causes poor social interaction and hospitalization. But, there is no information regards of the magnitude and contributing factors for aggressive behaviour among bipolar patients in Ethiopia. Therefore, this study was designed to assess the prevalence and associated factors of aggressive behaviour among patients with bipolar disorder.

**Method:**

An institutional based cross sectional study was conducted at Amanual Mental Specialized Hospital from May 1 to June 1, 2015 among 411 participants who were selected by systematic random sampling technique. Data was collected by interview technique by using Modified Overt Aggression Scale, entered and analyzed by using Epi Data 3.1 and Statistical Package for Social Science version 20, respectively. Adjusted Odd Ratio (AOR) with 95% Confidence Interval (CI) were used to show the odd and *P*-value <0.05 was considered as statistically significant.

**Results:**

A total of 411 bipolar patients were included in the study and the prevalence of aggressive behaviour was 29.4%. Significant associated factors for aggression were, having two or more episode [AOR = 2.35 95% CI (1.18, 4.69)], previous history of aggression, [AOR = 3.72, 95% CI (1.54, 8.98)], depressive symptoms [AOR = 3.63, 95% CI (1.89, 6.96)], psychotic symptoms [AOR = 5.41,95% CI (2.88, 10.1)], manic symptoms [AOR = 3.85,95% CI (2.06, 7.19)], poor medication adherence [AOR = 3.73 95% CI (1.71, 8.13)], poor social support [AOR = 2.99 95% CI (1.30, 6.91)] and current use of substance[AOR = 2.17 95% CI (1.16, 4.06)].

**Conclusion:**

Prevalence of aggression is high among bipolar patients and associated with many factors. So it needs public health attention to decrease aggression among bipolar patients.

## Background

Bipolar disorder is a severe and chronic psychiatric illness in which patients swing between episodes of depression and mania/hypomania that affects individual’s cognitive, emotion, behavior and social abilities [1]. Bipolar disorder has a 2.4% burden (years lived with disability) of world’s population that puts on the 6^th^ leading cause of disability-adjusted life years in aged 15–44 years. From 14–59% of bipolar patients has suicide ideation and 25–56% has at least one suicide attempt during lifetime, but 15–19% die from suicide. Even causes of suicidal behavior in bipolar disorder are multiple and complex, strong predictors link with impulsivity, aggression and hostility [2–4].

Aggression is a complex and heterogeneous behavior linked to violence, impulsivity, irritability, and hostility. Violence means physical attacks on other people, self, or objects while impulsivity is acting without control or premeditation. In other side, agitation means hostile or violent behavior or attitudes; readiness to attack or confront and hostility means unfriendly or threatening behavior or feelings [5].

Aggressive behavior is common among bipolar disorder patients that cause hospitalization especially during manic, mixed episodes, or psychotic states of bipolar disorders. Rate of violent crime is higher than the general population among bipolar patients and face to severe disruptions of occupational, societal, familial and other social functions [6].

Study from Czech Republic show that statistically significant increment for risk of violence among patients with bipolar disorder compare with general population. Even risk of violence was greater among patients with bipolar disorder than schizophrenic patients [7]. A number of studies were conducted regarding aggression among bipolar patients around the world; a systematic review of articles from 11 western countries revealed that the prevalence of aggression was 32.4% [8]. Another Meta analysis of 375 studies from Czech Republic and Slovakia indicates that the prevalence was 31.2% [9]. In Swedish national cohort study the prevalence of aggression was 22.2% [10]. Australian 18 month follow up study indicates that 28.6% show aggressive behaviour [11]. Cross sectional study in Nigeria showed that 24.5% of bipolar patient were aggressive [12]. Not only being bipolar patient but also, being male [13], having current psychotic symptoms [14], co-morbid substance abuse and personality disorders [15, 16] show significant association with aggressive behaviour. Trait aggression was significantly associated with diagnoses of childhood emotional abuse and neglect, childhood physical abuse, post-traumatic stress disorder, antisocial personality disorder [17].

There is a significance association between stress and psychiatric disorders that are predominantly known by aggression manifestation. In Italy, the absence of seclusion or physical restraint was associated with low rate of violence. By using modified version of the Morrison’s scale among Italian psychiatric in-patients, which was only 3.0% of the total 2395 admitted cases were violence in which physical restrain was absent [18, 19].

Systematic Review and Meta-Regression Analysis of 110 Studies in UK show that among 45,533 severe mental ill patients 8,439 (18.5%) were violent and significantly associated with non-adherence for psychological therapies and medication [20].

Despite this higher prevalence, there is no published data in Ethiopia regarding to aggression among bipolar patients. Therefore, this study was intended to assess the prevalence of aggression and associated factors among bipolar patients who attending Amanual mental specialized hospital, Addis Ababa, Ethiopia.

## Methods

### Study setting

Institution based cross sectional study was conducted among bipolar patients who were attending at Amanual Mental Specialized Hospital outpatient clinic. Amanual Mental Specialized Hospital is the only mental hospital in the country and has 15 outpatient departments, among these 8 outpatient departments serves an average of 11,760 bipolar follow-up patients per year and other mentally ill patients. Amanual Mental Specialized Hospital has 300 beds that used to inpatient service for psychiatric patients including bipolar cases.

### Participants

All patients who were clinically diagnosis (with Diagnostic and Statistical Manual of Mental Disorders IV^th^ edition), by psychiatrists and mental health professional specialists (masters holder on mental health) as bipolar disorder (any type of bipolar disorder) at Amanual Mental Specialized Hospital were the source population. Study population was patients who were attending at outpatient departments during data collection period and those included in the sample. Patients with bipolar disorder and whose age 18 years and above were included in the study and those who were seriously ill and unable to communicate were not included in the study.

The number of sample required for the study was estimated by taking 50% of the prevalence of aggression among bipolar patients in a single population proportion for unknown prevalence, with 5% of marginal error and 10% non response rate. Systematic random sampling technique used for a total 411 participants with every two cases to selected representative sample. Data was collected by degree holder psychiatric nurses with interviewing clients by using a semi-structured questionnaire, from May 1 to June 1, 2015.

### Instrument

The presence of aggressive behavior among bipolar patients in the last 7 days was assessed by using the Modified Overt Aggression Scale (MOAS) [21]. MOAS has a total of 16 items with four categories that assesses aggression, namely verbal aggression (with 4 items), aggression against property (with 4 items), auto-aggression (with 4 items), and physical aggression (with 4 items). The severity of aggressive behaviour ranges from 0 (no aggression) to 4 points (maximum violence) for each category. MOAS is validated in Africa, Nigeria [22] among psychiatric patients and has internal consistency 0.84.

Drug adherence was assessed by using Morsiky Medication Adherence Scale 8 item tool [23] and social support was assessed by using Oslo-3 Social support Scale [24]. Perceived stress was assessed by using of Perceived Stress Scale 10 items tool [25] that focus patients experienced stressful life events in the last one month and current substance uses was assessed by adopted alcohol smoking and substance involvement screening test [26].

### Analysis

The data was entered and clean up with Epi Data 3.1 and analyzed by using Statistical Package for Social Science version 20. Descriptive statistic was used to show clear picture of the problem. Multivariate and binary logistic regression analysis was implemented to identify factors associated with aggressive behaviour. The strength of the association was presented by odds ratio with 95% confidence interval (CI). *P* value less than 0.05 were considered as statistically significant in this study.

### Ethical clearance

Ethical approval was obtained from Ethical Review Board of University of Gondar and Amanual Mental Specialized Hospital. Formal permission letters was taken from administrative of the hospital and written consent taken from the participants for their voluntary participation. Confidentiality is maintained by omitting personal identification.

## Results

A total of 411 bipolar patients were included in the study, 175 (42.6%) were male and 236 (57.4%) were females. The median age of participants was 32 years with inter quartile range 14 and most of the participants 294 (71.5%) were from urban (Table 1).

**Table 1:** distribution of bipolar patients (*n* = 411) by socio-demographic factors related to aggressive behaviour attending at Amanual Mental Specialized Hospital; May, 2015

	Aggressive behavior			
Characteristics	Yes	No	Overall	Chisq test(X^2^)
Sex				
Male	49(28%)	126(72%)	175(42.6%)	0.30
Female	72(30.5%)	164(69.5%)	236(57.4%)	
Residency				
Urban	91(31%)	203(69%)	294(71.5%)	1.14
Rural	30(25.6%)	87(74.4%)	117(28.5%)	
Age				
18–26	33(31.4%)	72(68.6%)	105(25.55%)	2.73
27–32	29(26.9%)	79(73.1%)	108(26.3%)	
33–40	36(34.3%)	69(65.7%)	105(25.55%)	
41–75	23(24.7%)	70(75.3%)	93(22.6%)	
Education				
uneducated	14(20.3%)	55(79.7%)	69(16.8%)	3.69
Primary	43(31.4%)	94(68.6%)	137(33.33%)	
Secondary	40(30.3)	92(31.7%)	132(32.11%)	
Diploma	14(35%)	26(65%)	40(9.73%)	
Degree and above	10(30.3%)	23(69.7%)	33(8.03%)	
Religion				
Orthodox	80(32%)	170(68%)	250(60.8%)	5.08
Muslim	26(26%)	74(74%)	100(24.3%)	
Protestant	15(24.6%)	46(75.4%)	61(14.9%)	
Marital status				
Single	62(32.3%)	130(67.7%)	192(46.7%)	6.70
Married	45(25%)	135(75%)	180(43.8%)	
Divorced/separate/widowed	14(35.9%)	25(64.1%)	39(9.5%)	
Ethnicity				
Amahara	45(31.7%)	97(68.3%)	142(34.6%)	3.61
Oromo	36(26.7%)	99(73.3%)	135(32.8)	
Gurage	26(31.7%)	56(68.3%)	82(20%)	
Tigray	9(37.5%)	15(62.5%)	24(5.8%)	
Others ^a^	5(17.9%)	23(82.1%)	28(6.8%)	
Occupation				
Government employee	12(25.5%)	35(74.5%)	47(11.44%)	5.36
Nongovernmental employee	10(27.8%)	26(72.2%)	36(8.8%)	
Farmer	10(26.3%)	28(73.7%)	38(9.24%)	
Merchant	20(33.3%)	40(66.7%)	60(14.6%)	
Student	11(42.3%)	15(57.7%)	26(6.3%)	
Housewife	15(23.8%)	48(76.2%)	63(15.32%)	
Daily worker	4(20%)	16(80%)	20(4.9%)	
Jobless	39(32.2%)	82(67.8%)	121(29.4%)	
Living condition				
With family	104(29%)	255(71%)	359(87.3%)	30
Alone	17(32.7%)	35(67.3%)	52(12.7%)	
Wealth index				
Lowest	27(32.9%)	55(67.1%)	82(20%)	2.48
Second	27(30.7%)	61(69.3%)	88(21.4%)	
Middle	20(26%)	57(74%)	77(18.7%)	
Fourth	29(32.6%)	60(67.4%)	89(21.7%)	
Highest	18(24%)	57(76%)	75(18.2%)	

Others ^a^ (Wolaita, Sidama, Gamo)

### Clinical factors

Regarding to clinical factors of the participants 332 (80.8%) of the samples had history of aggression while 292 (61%) of the respondents had history of more than one episode of bipolar disorder (Table 2).

**Table 2:** distribution of bipolar patients by factors related to aggressive behaviour attending at Amanual Mental Specialized Hospital; May, 2015

	Aggressive behavior			
Characteristics	Yes	No	Overall	Chisq test (X^2^)
History of aggression				
Yes	110(33.1%)	222(66.9%)	332(80.8%)	11.34
No	71(13.9%)	68(86.1%)	79(19.2%)	
Number of episode				
One episode	19(16%)	100(84%)	119(29%)	14.64
More than one episode	102(34.9%)	190(65.1%)	292(71%)	
Current medication				
Antipsychotic				
Yes	71(28.7%)	176(71.3%)	247(60.1%)	.14
No	50(30.5%)	114(69.5%)	164(39.9%)	
Antidepressant				
Yes	15(26.3%)	42(73.7%)	57(13.9%)	.311
No	106(29.9%)	248(70.1%)	354(86.1%)	
Mood stabilizer				
Yes	95(30.4%)	218(69.6%)	313(76.5%)	.52
No	26(26.5%)	72(73.5%)	98(23.5%)	
Antipsychotic& mood stabilizer				
Yes	4(26.7%)	11(73.3%)	15(3.6%)	.06
No	117(29.5%)	279(70.5%)	396(96.4%)	
Adherence				
Poor	69(59%	48(41%)	117(28.5%)	69.40
Moderate	33(19.6%)	135(80.4%)	168(40.9%)	
High	19(15.1)	107(84.9%)	126(30.6%)	
Perceived stress				
Low	31(26.3%)	87(73.7%)	118(28.7%)	.98
Average	24(28.9%)	59(71.1%)	83(20.2%)	
High	66(31.4%)	144(68.6%)	210(51.1%)	
Social support				
Poor	53(37.3%)	89(62.7%)	142(34.5%)	7.00
Moderate	49(26.6%)	135(73.4%)	184(44.8%)	
Strong	19(22.4%)	66(77.6%)	85(20.7%)	
Family related problem				
Yes	62(40%)	93(60%)	155(37.7%)	13.36
No	59(23%)	197(77%)	256(62.3)	
Current substance use				
Yes	50(42.7%)	67(57.3%)	117(28.5%)	13.92
No	71(24.1%)	223(75.9%)	294(71.5%)	
Current symptoms				
Depressive				
Yes	93(47%)	105(53%)	198(48.2%)	56.51
No	28(13.1%	185(86.9%)	213(51.8%)	
Psychotic				
Yes	100(53.2%)	88(46.8%)	188(45.7%)	103.58
No	22(9.9%)	201(90.1%)	223(54.3%)	
Mania				
Yes	62(60.2%)	41(39.8%)	103(25.1%)	62.58
No	59(19.2%)	249(80.8%)	308(74.9%)	

In term of other psychiatric or neurological comorbidity 65 (15.8%) of the sample had co-morbid psychiatric or neurological disorders and 52.8% of the respondents had five or more years of morbidity.

### Magnitude of aggressive behaviour

Among the total 411 participants, 121 (29.4%) had aggressive behaviour. In terms of domain, verbal aggression was the highest which was account 84 (20.4%) of the total samples while auto aggression was the lowest that was 60 (14.6%) of the total sample. aggression against property was 71 (17.5%) and physical aggression was 78 (19.0%). As a combination, 30 (7.3%) of the sample had all domain of aggression.

Concerning to their clinical variables, of the total aggressive patients 90.9% of them had previous history of aggression, 66.1% had history of physical restrain, 51.1% had high perceived stress, 52.9% were on treatment more than five years, 78.5% of were on mood stabilizer, 58.7% of were on antipsychotic medication but, 57% of them had low adherence to their medication. In terms on their current symptom, among aggressive patients, 65.3% had guilty feeling, 61.2% depressed mood, 50.2% grandiosity, 54.5% suspicious and 40.0% had hallucination symptoms.

Among 121 aggressive bipolar patients 50 (41.3%) of them use any of these substances (alcohol, smoking and khat) currently.

### Multivariate analysis

After multivariate analysis of aggression in relation to all independent variables; number of episode, history of past aggression, psychotic symptoms, depressive symptoms, manic symptoms, low medication adherence, poor social support and current use of substance were found to be statistically significant (Table 3).

**Table 3:** factors associated with aggressive behaviour among patients with bipolar disorder attending at Amanual Mental Specialized Hospital; May, 2015

Explanatory variables	Aggressive behaviour Yes No	(Crude odd ratio) (95% CI)	Adjusted odd ratio (AOR) 95% CI
Number of episode			
One episode	19 100	1.00	1.00
More than one episode	102 190	2.82 (1.63, 4.87)*	2.35 (1.18, 4.69)**
History of aggression			
Yes	110 222	3.06 (1.55, 6.02)*	3.72 (1.54, 8.98)**
No	11 68	1.00	1.00
Co-morbid illness			
Yes	26 39	1.76 (1.02, 3.03)*	1.55 (0.74, 3.23)
No	95 251	1.00	1.00
Family related problems			
Yes	62 93	2.20 (1.44, 3.43)*	1.47 (0.82, 2.66)
No	59 197	1.00	1.00
Manic symptoms			
Yes	62 41	6.38 (3.93, 10.37)*	3.85 (2.06, 7.19)$
No	59 249	100	1.00
Depressive symptoms			
Yes	93 105	5.85 (3.60, 9.51)*	3.63 (1.89, 6.96)$
No	28 185	1.00	1.00
Psychotic symptoms			
Yes	100 88	10.93 (6.41, 18.62)*	5.41 (2.88, 10.1)$
No	21 202	1.00	1.00
Adherence			
Poor adherence	69 48	8.09 (4.39, 14.92)*	3.73 (1.71, 8.13)**
Moderate adherence	33 135	1.38 (0.74, 2.55)*	1.45 (0.67, 3.12)
Good adherence	19 107	1.00	1.00
Current use of substance			
Yes	50 67	2.34 (1.49, 3.69)*	2.17 (1.16, 4.06)**
No	71 223	1.00	1.00
Social support			
Poor	53 87	2.07 (1.12, 3.82)*	2.99 (1.30, 6.91)**
Moderate	49 135	1.26 (0.69, 2.31)*	1.56 (0.70, 3.47)
Strong	19 66	1.00	1.00

*p-value < 0.05

**statistically significant at multivariate logistic regression

$*p*-value <0.001

## Discussion

In psychopathology, aggression is the predominant symptom of many psychiatric disorders and aggressive behaviour is responsible for worsening of the morbidity of bipolar patients that cause hospitalization and poor social interaction.

The prevalence is almost similar with the study done in Australia which was 28.6% [11] but slightly higher than study results from Swedish and Nigeria, 22.2% [10], 24.5% [12] respectively. For this discrepancy; factors like methodological differences, clinical and other psycho-social factors of the study participants might be responsible.

After multivariate binary logistic regression analysis, those participants who had two or more episode had increase the risk of aggression by two times than had only one episode [AOR = 2.35 95% CI (1.18, 4.69)], which is in line with a study done in Sweden [10].

Patients who had history of past aggression were more than three times more likely to be aggressive as compared with those hadn’t past aggressive behaviour with odds of [AOR = 3.72, 95% CI (1.54, 8.98)], which coincide with Australian [11] and Sweden study [10]. This may be due to the relation between being aggressive and the expectation by experiences of this behaviour in the society or environment.

Clinical factors like, patients with depressive symptoms had more than three times likely had aggressive behaviour than those patients without depressive symptoms [AOR = 3.63, 95% CI (1.89, 6.96)], patients with psychotic symptoms had five times more likely had aggressive behaviour than patients without psychotic symptoms [AOR = 5.41, 95% CI (2.88, 10.13)]. Patients with mania symptoms had more than three times more likely aggressive behaviour than patients without mania symptoms [AOR = 3.85, 95% CI (2.06, 7.19)]. This finding was in harmonious with American study [14], Nigerian study for psychotic symptoms [12] and Australian [11] study for psychotic and mania symptoms.

Regarding to their medication adherence, patients who had low adherence to their medication had more than 3 times more likely had aggressive behaviour than patients who had high adherence [AOR = 3.73, 95% CI (1.71, 8.13)], which was in line with a study done in UK and Czech Republic that medication non-adherence was significantly associated with violent among severely mental ill patients [20, 27]. The possible explanation for this may be patients had poor adherence may not got good control for their symptoms.

Any current substance usage of (alcohol, smoking, and khat) among bipolar patients had two times more likely aggressive than patients who hadn’t use of these substances [AOR = 2.17, 95% CI (1.16, 4.06)]. Studies in Sweden, Nigeria, USA, and Czech Republic agree with this finding in which use of substances increase the impulsivity and emotionality of bipolar patients [10, 12, 17, 27]. The possible justification may be use of these substances had influence on the effect of medication and worsening of active symptoms.

Not only in patients but also in the general population use of substance like Khat shown in high proportion among individuals who show anger expression. Khat has the contributing effect for committing gender-based violent acts among general population [28, 29].

Regarding to their social support those bipolar patients who had poor social support were three times more likely had aggressive behaviour than had strong support supports [AOR = 2.99, 95% CI (1.30, 6.91)] and that may be due to lack of good care provider, attention giver that should upset the patient and ending up with aggressiveness for the surroundings.

## Conclusion

Prevalence of aggressive behaviour is high among bipolar patients who attending at Amanuel Mental specialized hospital outpatient clinic. Number of episode, history of past aggression, and presence of psychotic, depressive or manic symptoms, medication adherence, social support and current use of substance were found to be significantly associated with aggressive behaviour. It is important to pay attention for those significant factors to manage and control aggressive behaviour among bipolar patients.

### Limitation

This study was a cross-sectional study design that cannot show the temporal cause-effect association between factors and aggressive behaviour. Even though, each substance has its own contribution on aggressive behaviour, in this study type of substance were not assess whether it has association or has not with aggressive behaviour. This study also didn’t assess personality disorder among bipolar patients. We use the term bipolar disorder for all bipolar type (bipolar I, II, cyclothymia and others) and aggressive behaviour may different in each sub-type.

## Abbreviations

AOR: Adjusted odd ratio
CI: Confidence interval
MOAS: Modified overt aggression scale
UK: United Kingdom
USA: United State of America

## References

[1] American Psychiatric Association. Diagnostic and statistical manual of mental disorders, (DSM-5®). Arlington: American Psychiatric Pub; 2013.

[2] Mathers C, Boerma T, Ma Fat D (2008). The global burden of disease: 2004 update.

[3] Abreu LN, Lafer B, Baca-Garcia E, Oquendo MA (2009). Suicidal ideation and suicide attempts in bipolar disorder type I: an update for the clinician. Rev Bras Psiquiatr.

[4] Dalton EJ, Cate‐Carter TD, Mundo E, Parikh SV, Kennedy JL (2003). Suicide risk in bipolar patients: the role of co‐morbid substance use disorders. Bipolar Disord.

[5] Comai S, Tau M, Gobbi G (2012). The psychopharmacology of aggressive behavior: a translational approach: part 1: neurobiology. J Clin Psychopharmacol.

[6] Latalova K (2009). Bipolar disorder and aggression. Int J Clin Pract.

[7] Volavka J (2013). Violence in schizophrenia and bipolar disorder. Psychiatr Danub.

[8] Bowers L, Stewart D, Papadopoulos C, Dack C, Ross J, Khanom H (2011). Inpatient violence and aggression: a literature review.

[9] Nawka A, Rukavina TV, Nawková L, Jovanović N, Brborović O, Raboch J. Psychiatric disorders and aggression in the printed media: is there a link? a central European perspective. BMC psychiatry. 2012;12(1):19. 10.1186/1471-244X-12-19 PMC3352122 22409957

[10] Webb RT, Lichtenstein P, Larsson H, Geddes JR, Fazel S. Suicide, hospital- presenting suicide attempts, and criminality in bipolar disorder: examination of risk for multiple adverse outcomes. J Clin Psychiatry. 2014;75(8):809-16. 10.4088/JCP.13m08899 PMC4226039 25191918

[11] Barlow K, Grenyer B, Ilkiw‐Lavalle O (2000). Prevalence and precipitants of aggression in psychiatric inpatient units. Aust N Z J Psychiatry.

[12] Amoo G, Fatoye FO. Aggressive behaviour and mental illness: a study of in- patients at Aro Neuropsychiatric Hospital, Abeokuta. Niger J Clin Pract. 2010;13(3):351-55. 20857802

[13] Krakowski M, Czobor P (2004). Gender differences in violent behaviors: relationship to clinical symptoms and psychosocial factors. Am J Psychiatry.

[14] Strakowski SM, Fleck DE, DelBello MP, Adler CM, Shear PK, Kotwal R (2010). Impulsivity across the course of bipolar disorder. Bipolar Disord.

[15] Grunebaum MF, Galfalvy HC, Nichols CM, Caldeira NA, Sher L, Dervic K (2006). Aggression and substance abuse in bipolar disorder. Bipolar Disord.

[16] Swann AC, Dougherty DM, Pazzaglia PJ, Pham M, Steinberg JL, Moeller FG (2005). Increased impulsivity associated with severity of suicide attempt history in patients with bipolar disorder. Am J Psychiatr.

[17] Garno JL, Gunawardane N, Goldberg JF (2008). Predictors of trait aggression in bipolar disorder. Bipolar Disord.

[18] Ahmad A, Mazlan NH (2012). The kind of mental health problems and it association with aggressiveness: a study on security guards. Int JPsychol Behav Sci.

[19] Raja M, Azzoni A. Hostility and violence of acute psychiatric inpatients. Clin Pract Epidemiol Ment Health. 2005;1(1):1. 10.1186/1745-0179-1-11 PMC1188062 16053528

[20] Witt K, Van Dorn R, Fazel S. Risk factors for violence in psychosis: systematic review and meta-regression analysis of 110 studies. PloS one. 2013;8(2):e55942. 10.1371/journal.pone.0055942 PMC3572179 23418482

[21] Kay SR, Wolkenfeld F, Murrill LM (1988). Profiles of aggression among psychiatric patients: II. Covariates and predictors. J Nerv Ment Dis.

[22] Chukwujekwu DC, Stanley PC (2008). The modified overt aggression scale: how valid in this environment?. Niger J Med.

[23] Morisky DE, Ang A, Krousel‐Wood M, Ward HJ (2008). Predictive validity of a medication adherence measure in an outpatient setting. J Clin Hypertens.

[24] Bøen H. Characteristics of senior centre users–and the impact of a group programme on social support and late-life depression. Norsk Epidemiol. 2012;22(2):261-69.

[25] Cohen S, Kamarck T, Mermelstein R. A global measure of perceived stress. J Health Soc Behav. 1983;24(4):385–96. 6668417

[26] Humeniuk R, Ali R, Babor TF, Farrell M, Formigoni ML, Jittiwutikarn J (2008). Validation of the alcohol, smoking and substance involvement screening test (ASSIST). Addiction.

[27] Stuart H. Violence and mental illness: an overview. World Psychiatry. 2003;2(2):121-24. PMC1525086 16946914

[28] Terasaki DJ, Gelaye B, Berhane Y, Williams MA (2009). Anger expression, violent behavior, and symptoms of depression among male college students in Ethiopia. BMC Public Health.

[29] Philpart M, Goshu M, Gelaye B, Williams MA, Berhane Y (2009). Prevalence and risk factors of gender-based violence committed by male college students in Awassa, Ethiopia. Violence Vict.

